# Payments from drug companies to physicians are associated with higher volume and more expensive opioid analgesic prescribing

**DOI:** 10.1371/journal.pone.0209383

**Published:** 2018-12-19

**Authors:** Mark A. Zezza, Marcus A. Bachhuber

**Affiliations:** 1 New York State Health Foundation, New York City, New York, United States of America; 2 Division of General Internal Medicine, Albert Einstein College of Medicine, Montefiore Medical Center, Bronx, New York, United States of America; University of Washington, UNITED STATES

## Abstract

**Background:**

While the rise in opioid analgesic prescribing and overdose deaths was multifactorial, financial relationships between opioid drug manufacturers and physicians may be one important factor.

**Methods:**

Using national data from 2013 to 2015, we conducted a retrospective cohort study linking the Open Payments database and Medicare Part D drug utilization data. We created two cohorts of physicians, those receiving opioid-related payments in 2014 and 2015, but not in 2013, and those receiving opioid-related payments in 2015 but not in 2013 and 2014. Our main outcome measures were expenditures on filled prescriptions, daily doses filled, and expenditures per daily dose. For each cohort, we created a comparison group that did not receive an opioid-related payment in any year and was matched on state, specialty, and baseline opioid expenditures. We used a difference-in-differences analysis with linear generalized estimating equations regression models.

**Results:**

We identified 6,322 physicians who received opioid-related payments in 2014 and 2015, but not in 2013; they received a mean total of $251. Relative to comparison group physicians, they had a significantly larger increase in mean opioid expenditures ($6,171; 95% CI: 4,997 to 7,346), daily doses dispensed (1,574; 95%CI: 1,330 to 1,818) and mean expenditures per daily dose ($0.38; 95% CI: 0.29 to 0.47). We identified 8,669 physicians who received opioid-related payments in 2015, but not in 2013 or 2014; they received a mean total of $40. Relative to comparison physicians, they also had a larger increase in mean opioid expenditures ($1,031; 95% CI: 603 to 1,460), daily doses dispensed (557; 95% CI: 417 to 697), and expenditures per daily dose ($0.06; 95% CI: 0.002 to 0.13).

**Conclusions:**

Our findings add to the growing public policy concern that payments from opioid drug manufacturers can influence physician prescribing. Interventions are needed to reduce such promotional activities or to mitigate their influence.

## Introduction

Between 1999 and 2015, opioid analgesic prescribing more than tripled [1]. In parallel, rates of misuse, use disorder, and overdose also increased [2–5]. Recently, heroin and fentanyls have overtaken prescription opioid analgesics as major drivers of the opioid epidemic; however, prescription opioid analgesics were still involved in approximately 40% of overdoses in 2015 [3, 6]. In addition to direct involvement in overdose deaths, use of prescription opioid analgesics is strongly linked with the use of illicit opioids [7, 8].

While the rise in opioid analgesic prescribing and overdose deaths was multifactorial, financial relationships between opioid drug manufacturers and physicians may be one important factor. In other areas of medicine, evidence has suggested that these financial relationships significantly influence prescribing [9–11]. Mounting public and policymaker concern about financial conflicts of interest led to the passage of the Physician Payments Sunshine Act (PPSA) in 2010, which required disclosure of such relationships, with few exceptions, starting in 2013. In addition, records of these financial relationships were released to the public in the Open Payments database maintained by the Centers for Medicare & Medicaid Services (CMS).

With the availability of data, financial relationships between opioid drug manufacturers and physicians proved to be common and have been associated with increased prescribing. In one study, approximately one in twelve U.S. physicians had accepted payments from opioid manufacturers between 2013 and 2015 for a promotional activity related to an opioid (such payments are hereafter termed “opioid-related payments”). In total, more than 68,000 physicians received about $46 million in non-research payments [12]. In a recent analysis of opioid prescribing for Medicare enrollees, compared with physicians who received no such payments, physicians receiving any opioid-related payment in 2014 had 9.3% more opioid analgesic claims in 2015 [13]. In a study of New York physicians that also examined opioid prescribing for Medicare enrollees, $10 in opioid-related payments was associated with an additional $100 in expenditures for dispensed opioids relative to a matched comparison group of physicians that did not receive such payments [14].

While these studies provide evidence that opioid-related payments can influence prescribing, previous research has several limitations including lack of a comparison group or limitation to physicians in one state. Using Open Payments data combined with Medicare prescription drug utilization data, we conducted a retrospective cohort study with a difference-in-differences framework to estimate the impact of opioid-related payments on opioid analgesic prescribing overall and by physician specialty. This study extends prior research by using data on physicians receiving opioid-related payments across the country and over time, along with a matched comparison group of physicians who did not receive payments. While causation cannot be definitively established from observational data, use of a strong observational design with a comparison group can help account for the fact that physicians receiving opioid-related payments are different than physicians that do not receive such payments. Beyond prescribing volume, we also examine total expenditures and expenditures per daily dose to determine if opioid-related payments are associated with prescribing of more expensive opioids. Finally, we analyze the possible dose-response relationship between opioid-related payments and prescribing, strengthening evidence of a causal relationship.

## Methods

### Data sources and elements

#### Open Payments

To identify all opioid-related payments, we used August 2013 through December 2015 Open Payments data released by October 26, 2017 [15]. The Open Payments data provide detailed information on each payment including the payment date, activity covered, amount, form of payment, and specific drug associated with the payment. Details on the physicians receiving the payments are also reported, including; name, specialty, and location. Since the reporting requirements began in August 2013, the data for 2013 only cover the second half of the year.

#### Medicare Provider Utilization and Payment Data

To identify physicians writing opioid analgesic prescriptions filled under Medicare Part D, we used the Medicare Provider Utilization and Payment Data: Part D Prescriber Files [16]. These files contain prescriber-level data including the total number of prescriptions written by the prescriber that were dispensed to patients in the given calendar year (original prescriptions and refills), the total daily doses dispensed for the drug, and the total expenditures for the medication. The total expenditures are based on the amount paid by the Medicare Part D plan and the Medicare enrollee, as well as by other third-party payers or government subsidies, and include the ingredient cost of the medication, dispensing fees, sales tax, and any applicable administration fees. To account for inflation, we adjusted expenditures to 2015 dollars using the prescription drug-specific Consumer Price Index [17, 18].

From the Medicare Provider Utilization and Payment Data, we also extracted each physician’s average CMS Hierarchical Condition Categories (CMS-HCC) risk score for the Medicare enrollees treated by each physician in each year [19]. The CMS-HCC risk score uses demographic information and diagnoses on Medicare FFS claims to measure each enrollee’s co-morbidities, with higher scores going to enrollees with more (or more severe) comorbidities. Hence, we used the risk scores as a proxy for health status.

### Inclusion and exclusion criteria

We first identified opioid-related payments, defined as payments from the Open Payment data that were linked to an opioid analgesic as defined by CMS [20]. We included direct and indirect monetary payments for promotional activities as well as payments in kind such as the value of food and gifts. We excluded research-related payments and information on equity stakes as these payments may include compensation for intellectual property, are less likely to target specific prescribing behaviors, and may be provided to physicians not primarily practicing clinical medicine. Because sublingual formulations of buprenorphine and buprenorphine/naloxone are primarily used for treatment of opioid use disorder, we also excluded payments related to these medications.

Next, we identified eligible physicians from Medicare prescribing data. We included only physicians with 10 or more filled opioid analgesic prescriptions in each calendar year, according to the Medicare Provider Utilization and Payment Data. We excluded all non-physician prescribers including those identified as institutional prescribers. We also excluded physicians with a missing CMS-HCC risk score in any year.

### Study population

After identifying opioid-related payments and eligible physicians, we match-merged the two data sources. As National Provider Identification numbers were explicitly forbidden by statute from release as part of the Open Payments program, we used a string-matching algorithm based on the provider names and locations on each data set. Matching was done first by requiring a match of first name, last name, middle initial, and ZIP code. A second, less stringent, attempt at matching was conducted with the physicians who were left unmatched from the first attempt, based on first name, last name, and ZIP code only. A similar approach has been used in previous work [21, 22].

To examine the association between opioid-related payments and prescribing, we identified two cohorts of physicians; 1) those who received opioid-related payments in 2014 and 2015, but not in 2013, and 2) those who received opioid-related payments in 2015, but not in 2013 or 2014. For each group, we then identified comparison physicians who did not receive payments in any year. To generate these comparison groups, we used coarsened exact matching and matched on characteristics that may influence opioid analgesic prescribing: state, specialty, and baseline opioid expenditures [23]. For specialty, we created strata for matching based on categories used by the National Ambulatory Medical Care Survey (S1 Table) [24]. Specialties that did not exist in both the eligible payment-receiving and comparison groups were excluded (and are not reflected in S1 Table).

For opioid expenditures, we created strata of spending based on percentile distributions and we matched on opioid expenditures in years prior to when opioid-related payments were made. For the cohort that received opioid-related payments in 2014 and 2015, but not 2013, we categorized physicians based on deciles of opioid expenditures in 2013. For the cohort that received opioid-related payments in 2015, but not 2013 or 2014, we similarly matched on 2013 opioid expenditures, as well 20 strata of 2014 opioid expenditures based on every 5^th^ percentile. Matching on both years helped ensure that the trajectory of opioid prescribing was similar in the baseline period. All percentile categories used for matching are based on the distributions of the relevant cohort of physicians that received an opioid-related payment.

We matched payment-receiving physicians to comparison physicians by state, specialty, and baseline opioid expenditures; comparison physicians needed an exact match on all variables to be included. We used weighting to account for any imbalance in the number of physicians receiving an opioid-related payment and comparison physicians in any strata [25]. The distribution of physicians across the state, specialty and baseline opioid expenditure strata pre- and post-matching are shown in S2A–S2D Table.

### Main outcome measures

For all dispensed opioid analgesic prescriptions from each physician, we analyzed three measures of opioid analgesic prescribing: 1) expenditures on filled prescriptions (hereafter referred to as opioid expenditures), 2) daily doses filled (i.e., days supply based on physicians’ dosing instructions), and 3) expenditures per daily dose. We calculated the physician- and year-specific expenditures per daily dose separately for each calendar year by dividing the total expenditures by the total daily doses dispensed.

### Main exposure

Our main exposure was receipt of any opioid-related payment in a given calendar year, measured in two ways. First, we classified opioid-related payments as dichotomous (yes/no in a given year). Next, for the cohort of physicians who received opioid-related payments 2014 and 2015, but not 2013, we also classified physicians into four subgroups depending on the amount of payments received in 2014 and 2015.

### Statistical analysis

We used a difference-in-differences approach. To account for outcome correlations within physicians over time, we used linear generalized estimating equations regression models with an exchangeable working correlation structure. The exchangeable working correlation was chosen as the correlation within physicians over time is expected to be high and the quasi information criterion was relatively low in models tested compared to other structures. For the two cohorts of physicians, we conducted separate regression models.

For the main models, we included a dichotomous variable to indicate which physicians received an opioid-related payment (i.e., payment-receiving versus comparison physicians), a dichotomous variable to indicate the year in which opioid-related payments were made, and an interaction between those group and time dichotomous variables. The coefficient on the interaction term provides the difference-in -differences estimate. To account for differences in medical comorbidities between physicians’ patient panels, we also included the average CMS-HCC risk score of the Medicare enrollees treated by each physician in each year as a covariate in the models.

To explore the association between the amount of opioid-related payments received and opioid prescribing, we also estimated regressions using four subgroups of physicians depending on the amount of payments received. We did this for the cohort of physicians that initially received a payment in 2014 and 2015, but not in 2013. The four payment amounts used are: physicians receiving less than the 33^rd^ percentile of payments ($45); greater than or equal to the 33^rd^ percentile and less than the 66^th^ percentile ($91); greater than or equal to the 66^th^ percentile and less than the 95^th^ percentile ($327); and equal to or above the 95^th^ percentile. There was a wide range of payments received by the physicians in the highest percentile group, which is why we assessed the top fifth percentile group separately. These regression models are similar to the main regression models; except, instead of one dichotomous variable indicating which physicians received opioid-related payments, there are four dichotomous variables representing each of the payment-amount subgroups. In addition, there are interaction terms between each of the payment-amount variables and the time dichotomous variable indicating when payments were received.

### Subgroup analyses

We performed a subgroup analysis to explore whether associations differed by specialty. For this analysis we excluded the specialty categories of dentistry, diagnostic radiology and interventional radiology, addiction medicine and psychiatry, hospital-based non-surgical, oncology surgical, and podiatry due to small sample sizes.

### Sensitivity analysis

The difference-in-differences framework relies on the assumption of “parallel trends”. For our analysis, this assumption requires that the pre-intervention trends in the outcome variables are similar for the group of physicians that received opioid-related payments and for the comparison group of physicians that did not. The coarsened extract matching approach used to select the comparison group helps ensure that levels of opioid expenditures in years prior to when opioid-related payments were made are similar for both groups. To further examine the parallel trends assumption, we also conducted a variant of the main regression for the cohort of physicians that received payments in 2015, but not 2013 and 2014, to test whether there were differences in the changes over time in any of the three outcome measures prior to when payments were received. A similar test was not possible for the cohort of physicians who received opioid-related payments in 2014 and 2015, but not in 2013, as only one year of pre-intervention data was available.

Also, to assess the degree to which our findings represent a specific relationship between opioid-related payments and opioid prescribing, as opposed to secular trends or contemporaneous exposures, we conducted a sensitivity analysis. Instead of expenditures related to opioid prescribing, we used expenditures related to antibiotic prescribing as the main outcome.

SAS version 9.4 (SAS Institute, Cary, NC) was used for all analyses.

## Results

There were 6,443 physicians that received an opioid-related payment in 2014 and 2015, but not in 2013, and had corresponding Medicare prescribing data. There were 9,529 physicians that received an opioid-related payment in 2015, but not in 2013 or 2014, and had corresponding Medicare prescribing data. After excluding non-physicians and physicians practicing in a state or with a specialty not matching a comparison physician, there were 6,432 and 9,503 physicians remaining, respectively. After conducting the matching procedure, which led to additional exclusions based on strata used for matching, there were 6,322 and 8,669 physicians, respectively, who received opioid-related payments.

Physicians who received opioid-related payments in 2014 and 2015, but not in 2013, received a mean of 6.6 payments during those two years, amounting to about a mean of $251 (Table 1). Physicians who received payments in 2015, but not in 2013 or 2014, received a mean of 2.2 payments totaling a mean of $40.

**Table 1:** Amount and number of payments from drug manufacturers to physicians for opioids: August 2013 –December 2015.

Year	Physicians that Received Opioid-Related Payments in 2014 and 2015, but not in 2013 (n = 6,322)				Physicians that Received Opioid-Related Payments in 2015, but not in 2013 and 2014 (n = 8,669)			
	Number of Payments	Total Payment Amount	Mean Number of Payments per Physician *(Standard Deviation)*	Mean Payment per Physician *(Standard Deviation)*	Number of Payments	Total Payment Amount	Mean Number of Payments per Physician *(Standard Deviation)*	Mean Payment per Physician *(Standard Deviation)*
**2013**	—	—	—	—	—	—	—	—
**2014**	15,857	$491,936	2.5 *(3*.*1)*	$79 *(840)*	—	—	—	—
**2015**	25,707	$1,097,271	4.1 *(7*.*0)*	$174 *(2*,*156)*	18,748	$344,958	2.2 *(2*.*6)*	$40 *(279)*
**2013–2015**	41,564	$1,589,208	6.6 *(8*.*9)*	$251 *(2*,*771)*	18,748	$344,958	2.2 *(2*.*6)*	$40 *(279)*

Note: The years may not add up to the totals due to rounding.

Relative to comparison group physicians, physicians who received an opioid-related payment in 2014 and 2015, but not in 2013, had a significantly larger increase in mean opioid expenditures ($6,171; 95% CI: 4,997 to 7,346), daily doses dispensed (1,574; 95%CI: 1,330 to 1,818) and mean expenditures per daily dose ($0.38; 95% CI: 0.29 to 0.47; Table 2).

**Table 2:** Association between opioid-related payments and Medicare Part D opioid prescribing for physicians that received payments in 2014 and 2015, but not in 2013.

	Mean Annual Expenditures for Dispensed Opioids Under Medicare Part D*^#^	Mean Annual Daily Doses for Dispensed Opioids Under Medicare Part D^#^	Mean Annual Expenditures per Daily Dose for Dispensed Opioids Under Medicare Part D* ^#^
**Payment-receiving physicians (n = 6,322)**			
Pre-Intervention Period (2013)	$30,444	13,524	$2.24
Post Intervention Period (2014 and 2015)	$38,468	14,971	$2.61
**Comparison physicians (n = 191,478)**			
Pre-Intervention Period (2013)	$30,191	12,865	$2.51
Post Intervention Period (2014 and 2015)	$32,043	12,738	$2.50
**Difference-in-differences estimate**	$6,171	1,574	$0.38
SE	*599*	*124*	*0*.*05*
95% CI	*(4*,*997–7*,*346)*	*(1*,*330–1*,*818)*	*(0*.*29–0*.*47)*
p-Value	*<0*.*0001*	*<0*.*0001*	*<0*.*0001*

Notes

* Adjusted for differences in prices over time ($ 2015).

# Adjusted for average risk scores of beneficiaries treated by each provider.

The cohort of physicians who received an opioid-related payment in 2015, but not 2013 or 2014, also had a significant increase in opioid expenditures ($1,031; 95% CI: 603 to 1,460), daily doses dispensed (557; 95% CI: 417 to 697), and expenditures per daily dose ($0.06; 95% CI: 0.002 to 0.13), over and above changes in the comparison group (Table 3).

**Table 3:** Association between opioid-related payments and Medicare Part D opioid prescribing for physicians that received payments in 2015, but not in 2013 and 2014.

	Mean Annual Expenditures for Dispensed Opioids Under Medicare Part D*^#^	Mean Annual Daily Doses for Dispensed Opioids Under Medicare Part D^#^	Mean Annual Expenditures per Daily Dose for Dispensed Opioids Under Medicare Part D* ^#^
**Payment-receiving physicians (n = 8,669)**			
Pre-Intervention Period (2013 and 2014)	$20,552	10,624	$1.91
Post Intervention Period (2015)	$21,910	10,906	$1.98
**Comparison physicians (n = 153,723)**			
Pre-Intervention Period (2013 and 2014)	$20,498	9,941	$2.12
Post Intervention Period (2015)	$20,826	9,666	$2.12
**Difference-in-differences estimate**	$1,031	557	$0.06
SE	*219*	*71*	*0*.*03*
95% CI	*(603–1*,*460)*	*(417–697)*	*(0*.*002–0*.*13)*
p-Value	*<0*.*0001*	*<0*.*0001*	*<0*.*0441*

Notes

* Adjusted for differences in prices over time ($ 2015).

# Adjusted for average risk scores of beneficiaries treated by each provider.

The magnitude of the changes in mean opioid expenditures, daily doses, and expenditures per daily dose, was greater for physicians receiving higher payments. Among physicians receiving opioid-related payments in 2014 and 2015, but not 2013, those receiving less than $45 had a significant increase in mean opioid expenditures ($902; 95% CI: 74 to 1,730), over and above changes in the comparison group (Table 4). The relative increases were much higher as payments increased: $2,090 (95% CI: 1,060 to 3,119) for physicians receiving between $45 and $91 in opioid-related payments; $8,796 (95% CI: 6,808 to 10,784) for physicians receiving between $91 and $327; and $52,307 (95% CI: 36,253 to 68,360) for physicians receiving at least $327. Similar patterns of higher increases being associated with higher payments occurred for mean annual daily dose and mean annual expenditures per daily dose.

**Table 4:** Association between various amounts of opioid-related payments and Medicare Part D opioid prescribing for physicians receiving payments in 2014 and 2015, but not in 2013.

	Mean Annual Expenditures for Dispensed Opioids Under Medicare Part D*^#^	Mean Annual Daily Doses for Dispensed Opioids Under Medicare Part D^#^	Mean Annual Expenditures per Daily Dose for Dispensed Opioids Under Medicare Part D* ^#^
**Payment-receiving physicians (<$45) (n = 2,119)**	** **	** **	** **
Pre-Intervention Period (2013)	$21,835	11,145	$2.02
Post Intervention Period (2014 and 2015)	$24,603	11,664	$2.15
**Payment-receiving physicians ($45 - $91) (n = 2,055)**			
Pre-Intervention Period (2013)	$28,288	12,993	$2.17
Post Intervention Period (2014 and 2015)	$32,244	13,767	$2.37
**Payment-receiving physicians ($91 - $327) (n = 1,829)**			
Pre-Intervention Period (2013)	$38,990	16,113	$2.45
Post Intervention Period (2014 and 2015)	$49,652	18,300	$2.82
**Payment-receiving physicians ($327+) (n = 319)**			
Pre-Intervention Period (2013)	$50,520	18,443	$2.66
Post Intervention Period (2014 and 2015)	$104,692	26,102	$5.57
**Comparison physicians (n = 153,723)**			
Pre-Intervention Period (2013)	$30,168	12,871	$2.51
Post Intervention Period (2014 and 2015)	$32,034	12,741	$2.50
**Difference-in-differences estimate (<$45)**	$902	649	$0.15
SE	*423*	*136*	*0*.*03*
95% CI	*(74–1*,*730)*	*(383–916)*	*(0*.*10–0*.*20*
p-Value	*0*.*0328*	*<0*.*0001*	*<0*.*0001*
**Difference-in-differences estimate ($45 - $91)**	$2,090	905	$0.21
SE	*525*	*125*	*0*.*05*
95% CI	*(1*,*060–3*,*119)*	*(661–1*,*149)*	*(0*.*11–0*.*31)*
p-Value	*<0*.*0001*	*<0*.*0001*	*<0*.*0001*
**Difference-in-differences estimate ($91 - $327)**	$8,796	2317	$0.38
SE	*1*,*014*	*278*	*0*.*05*
95% CI	*(6*,*808–10*,*784)*	*(1*,*773–2*,*862)*	*(0*.*27–0*.*49)*
p-Value	*<0*.*0001*	*<0*.*0001*	*<0*.*0001*
			
**Difference-in-differences estimate ($327+)**	$52,307	7,789	$2.93
SE	*8*,*191*	*1*,*016*	*0*.*75*
95% CI	*(36*,*253–68*,*360)*	*(5*,*797–9*,*781)*	*(1*.*46–4*.*39)*
p-Value	*<0*.*0001*	*<0*.*0001*	*<0*.*0001*

Notes

* Adjusted for differences in prices over time ($ 2015).

# Adjusted for average risk scores of beneficiaries treated by each provider.

Associations between opioid-related payments and prescribing varied by specialty (Tables 5 and 6). For the group of physicians receiving payments in 2014 and 2015, but not 2013, the categories of specialties in which payment-receiving physicians had the largest increase in expenditures, over and above the comparison group, were anesthesiology and pain management ($10,532; 95% CI: 728 to 20,336), neurology ($10,266; 95% CI: 1,818 to 18,713), and physical medicine & rehabilitation and sports medicine ($10,665; 95% CI: 2,524 to 18,805). A similar pattern was found for daily dose and expenditures per daily dose.

**Table 5:** Association between opioid-related payments and Medicare Part D opioid prescribing for physicians receiving payments in 2014 and 2015, but not in 2013, by Specialty.

Specialty	Number of Physicians (unweighted)	Annual Expenditures for Dispensed Opioids Under Medicare Part* ^#^					Annual Daily Doses for Dispensed Opioids Under Medicare Part ^#^					Annual Expenditures per Daily Dose for Dispensed Opioids Under Medicare Part* ^#^				
		Mean		DiD	*SE*	*p-value*	Mean		DiD	*SE*	*p-value*	Mean		DiD	*SE*	*p-value*
		2013	2014/ 2015	*(95% CI)*			2013	2014/ 2015	*(95% CI)*			2013	2014/2015	*(95% CI)*		
**Anesthesiology and Pain Management**																
Payment-receiving physicians	420	$80,763	$110,648	$10,532	*5*,*002*	*0*.*0353*	27,894	34,735	2,620	*1*,*190*	*0*.*0276*	$2.96	$3.41	0.41	*0*.*13*	*0*.*0019*
Comparison physicians	1,540	$79,251	$98,604	*(728–20*,*336)*			26,216	30,437	*(288–4*,*952*			$3.19	$3.22	*(0*.*15–0*.*67)*		
**Neurology**																
Payment-receiving physicians	236	$25,581	$36,591	$10,266	4,310	*0*.*0172*	10,454	11,977	1,378	*642*	*0*.*0318*	$2.42	$2.75	0.42	*0*.*13*	*0*.*0017*
Comparison physicians	2,454	$29,430	$30,174	*(1*,*818–18*,*713)*			8,674	8,819	*(120–2*,*636)*			$3.12	$3.03	*(0*.*16–0*.*69)*		
**Non-Oncology Medical Specialty**																
Payment-receiving physicians	248	$27,686	$31,400	$3,687	1,256	*0*.*0033*	15,509	16,033	1,536	*346*	*<0*.*0001*	$1.93	$2.17	0.34	*0*.*12*	*0*.*0050*
Comparison physicians	7,469	$31,094	$31,120	*(1*,*225–6*,*149)*			14,692	13,679	*(858–2*,*214)*			$3.09	$2.98	*(0*.*10–0*.*58))*		
**Non-Oncology Surgical Subspecialty**																
Payment-receiving physicians	360	$8,297	$12,488	$4,282	1,411	*0*.*0024*	4,354	5,301	1,152	*391*	*0*.*0032*	$1.82	$2.19	0.19	*0*.*09*	*0*.*0363*
Comparison physicians	43,758	$9,514	$9,424	*(1*,*516–7*,*049)*			4,330	4,126	*(386–1*,*917)*			$2.20	$2.37	*(0*.*01–0*.*38)*		
**Oncology Medical Specialty**																
Payment-receiving physicians	310	$10,285	$18,723	$7,856	3,542	*0*.*0266*	2,945	3,245	253	*104*	*0*.*0149*	$3.35	$5.76	2.24	*0*.*77*	*0*.*0036*
Comparison physicians	5,299	$9,963	$10,545	*(913–14*,*799)*			2,732	2,779	*(49–457)*			$3.55	$3.71	*(0*.*73–3*.*75)*		
**Physical Medicine & Rehabilitation and Sports Medicine**																
Payment-receiving physicians	257	$55,558	$71,736	$10,665	4,153	*0*.*0102*	17,827	21,567	2,863	*1*,*022*	*0*.*0051*	$2.77	$3.06	0.20	*0*.*12*	*0*.*1019*
Comparison physicians	2,017	$59,199	$64,712	*(2*,*524–18*,*805)*			17,712	18,590	*(859–4867)*			$3.15	$3.25	*(-0*.*04–0*.*43)*		
**Primary Care**																
Payment-receiving physicians	4,392	$27,986	$33,456	$5,097	485	*<0*.*0001*	13,471	14,354	1,441	*101*	*<0*.*0001*	$2.08	$2.30	0.26	*0*.*03*	*<0*.*0001*
Comparison physicians	116,737	$27,192	$27,565	*(4*,*146–6*,*048)*			12,875	12,316	*(1*,*244–1*,*639)*			$2.30	$2.26	*(0*.*20–0*.*32)*		

Notes

* Adjusted for differences in prices over time ($ 2015).

# Adjusted for average risk scores of beneficiaries treated by each provider.

**Table 6:** Association between opioid-related payments and Medicare Part D opioid prescribing for physicians receiving payments in 2015, but not in 2013 and 2014, by Specialty.

Specialty	Number of Physicians (unweighted)	Annual Expenditures for Dispensed Opioids Under Medicare Part* ^#^					Annual Daily Doses for Dispensed Opioids Under Medicare Part ^#^					Annual Expenditures per Daily Dose for Dispensed Opioids Under Medicare Part* ^#^				
		Mean		DiD	*SE*	*p-value*	Mean		DiD	*SE*	*p-value*	Mean		DiD	*SE*	*p-value*
		2013/ 2014	2015	*(95% CI)*			2013/ 2014	2015	*(95% CI)*			2013/ 2014	2015	*(95% CI)*		
**Anesthesiology and Pain Management**																
Payment-receiving physicians	188	$102,415	$116,287	$2,219	*5*,*197*	*0*.*6694*	35,596	41,201	2,509	*1*,*905*	*0*.*1879*	$2.98	$3.25	0.28	*0*.*27*	*0*.*3011*
Comparison physicians	751	$99,062	$110,714	*(-7967–12*,*406)*			33,254	36,350	*(-1*,*226–6*,*244)*			$3.10	$3.09	*(-0*.*25–0*.*82)*		
**Neurology**																
Payment-receiving physicians	457	$7,940	$7,812	-$774	557	*0*.*1644*	3,473	3,383	-24	*156*	*0*.*8778*	$1.50	$1.55	-0.04	*0*.*07*	*0*.*5203*
Comparison physicians	1,588	$7,424	$8,071	*(-1*,*866–317)*			3,065	2,999	*(-331–283)*			$1.81	$1.90	*(-0*.*18–0*.*09)*		
**Non-Oncology Medical Specialty**																
Payment-receiving physicians	328	$17,034	$16,695	-$468	909	*0*.*6068*	10,820	10,410	86	*247*	*0*.*7289*	$1.36	$1.39	0.01	*0*.*14*	*0*.*9418*
Comparison physicians	4,293	$15,886	$16,014	*(-2*,*250–1*,*314)*			9,256	8,760	*(-399–570)*			$2.48	$2.50	*(-0*.*26–0*.*30)*		
**Non-Oncology Surgical Subspecialty**																
Payment-receiving physicians	829	$4,516	$5,388	$877	329	*0*.*0076*	2,480	2,693	315	*106*	*0*.*0030*	$1.89	$1.97	0.03	*0*.*04*	*0*.*3998*
Comparison physicians	34,341	$4,438	$4,433	*(233–1*,*521)*			2,527	2,424	*(107–524)*			$1.95	$1.99	*(-0*.*04–0*.*10)*		
**Oncology Medical Specialty**																
Payment-receiving physicians	461	$9,355	$11,477	$1,278	1,053	*0*.*2248*	2,582	2,734	110	*62*	*0*.*0749*	$3.66	$4.28	0.42	*0*.*51*	*0*.*4169*
Comparison physicians	2,081	$8,764	$9,608	*(-785–3*,*341)*			2,476	2,518	*(-11–230)*			$3.48	$3.68	*(-0*.*59–1*.*42)*		
**Physical Medicine & Rehabilitation and Sports Medicine**																
Payment-receiving physicians	191	$35,857	$39,134	$1,057	2,182	*0*.*628*	12,541	14,123	1,125	*678*	*0*.*0972*	$2.29	$2.45	0.19	*0*.*19*	*0*.*3164*
Comparison physicians	983	$41,795	$44,015	*(-3*,*219–5*,*333)*			12,935	13,392	*(-204–2453)*			$2.67	$2.63	*(-0*.*18–0*.*55)*		
**Primary Care**																
Payment-receiving physicians	6,027	$22,207	$23,255	$1,238	230	*<0*.*0001*	12,302	12,416	621	*75*	*<0*.*0001*	$1.77	$1.79	0.03	*0*.*02*	*0*.*0588*
Comparison physicians	92,530	$22,142	$21,951	*(788–1*,*689)*			11,474	10,968	*(473–768)*			$1.98	$1.97	*(0*.*001–0*.*07)*		

Notes

* Adjusted for differences in prices over time ($ 2015).

# Adjusted for average risk scores of beneficiaries treated by each provider.

In contrast, for the group of physicians receiving payments in 2015, but not in 2013 or 2014, there were only two specialty categories in which there was a significant relative increase in opioid expenditures over and above the change for comparison physicians. Primary care physicians who received payment had expenditures that were $1,238 (95% CI: 788 to 1,689) over and above any changes in the comparison group. Non-oncology medical specialty physicians had a relative increase of $877 (95% CI: 233 to 1,521). These specialties also had significant increases in the daily doses for dispensed opioids relative to comparison physicians. There were no significant relative changes in the expenditures per daily dose within specialty categories.

Results of the sensitivity test for parallel trends support the assumption that the trends in the outcome measures were similar in the pre-intervention period for the group of physicians that received opioid-related payments and the comparison group physicians (S3 Table).

Results of the sensitivity analysis to assess the degree to which our findings represent a specific relationship between opioid-related payments and opioid prescribing, as opposed to secular trends or contemporaneous exposures, were mixed. Unlike the trend in opioid expenditures, the trend in antibiotic expenditures was declining over time for both cohorts analyzed. However, when examining antibiotic prescribing, relative to comparison group physicians, there was a lesser decline in expenditures for antibiotics for the physicians that received opioid-related payments. Relative to comparison physicians, this resulted in a significant increase in antibiotic expenditures for the payment-receiving physicians in 2014 and 2015, but not in 2013 (S4 Table). Relative to comparison group physicians, we did not find a significant difference in antibiotic prescribing for physicians receiving an opioid-related payment in 2015, but not in 2013 or 2014.

## Discussion

In a national cohort of physicians taking care of Medicare-insured patients, we found that receiving opioid-related payments was associated with significantly increased opioid expenditures, daily doses dispensed, and expenditures per daily dose. We also found that a higher payment amount was associated with a larger increase in expenditures, daily doses, and expenditures per daily dose. Our findings add to growing concern that opioid-related payments from drug manufacturers directly increase opioid analgesic prescribing.

While prior studies have found associations between opioid-related payments and higher opioid prescribing, they have been largely cross-sectional or have not controlled for key differences between physicians receiving payments versus those that did not [13, 22]. Hence, it was not clear whether physicians were prescribing more after receiving payments, or if drug manufacturers were simply targeting higher-prescribing physicians. The one study that used a comparison group was limited to physicians in one state and did not use any statistical tests to determine if there were significant changes in opioid prescribing [14]. By using a comparison group with national data, our study moves the body of research closer to the direction of showing a causal relationship. Our finding of a dose-response relationship also strengthens the evidence for a causal relationship. By also looking at effects related to daily doses dispensed and expenditures per daily dose, as well as total opioid expenditures, we are able to provide some insight on whether physicians are prescribing more drugs and or more expensive drugs after receiving payments from the drug industry.

While there was a significant relative increase in opioid prescribing for physicians receiving less than $45, the estimated magnitude of the increases were substantially higher for physicians that received more money. Furthermore, the cohort of physicians that received opioid-related payments in 2014 and 2015, but not in 2013, received higher opioid-related payments on average than the cohort that received opioid-related payments in 2015, but not in 2013 or 2014. Consistent with the dose-response relationship, the increases in opioid prescribing were also generally higher for the cohort that received opioid-related payments in 2014 and 2015, but not in 2013.

This is also the first study to explore changes in both the quantity of opioids prescribed and in opioid expenditures after receiving opioid-related payments. We find that opioid-related payments are associated with both an increase in prescribed opioids, as well as a shift to more expensive opioids (i.e., higher expenditure per daily dose). These results were largely consistent across specialties, although the magnitude appears to have decreased over time with many of the changes not being significant for several of specialties in the cohort of physicians that received payments in 2015, but not in 2013 or 2014.

While we examined only the direct associations between opioid-related payments and prescribing, opioid-related payments may have spillover effects in two ways. First, opioid-related payments may lead physicians to recommend increases in opioid prescribing among their professional networks. The degree to which this may happen is unclear and is a potential target for future research. Second, opioid-related payments may increase physicians’ prescribing of branded medications, or increase medication prescribing more generally. We found some potential evidence of this as, in one cohort of physicians, those receiving opioid-related payments also had relative increases in antibiotic expenditures. Further research is needed to examine how opioid-related payments affect physicians’ networks and their prescribing of other types of medications.

While a better understanding of the potential influence of opioid-related payments is essential, it may not be prudent to wait for additional research before moving forward with additional interventions. At a policy level, further regulations, such as a blanket ban on most physician-targeting promotional activities or requiring that physicians specifically opt-in to drug manufacturer outreach, could reduce opioid-related payments [26]. At a physician level, interventions such as “academic detailing” can improve physicians’ adherence to clinical guidelines and could potentially be adapted to counteract marketing by opioid manufacturers [27] [28]. Finally, at a patient level, one goal of the publicly-available Open Payments database is to allow patients to look up their physicians; however, the degree to which this occurs and patients subsequently understand and act on the information presented is unclear. Interventions to help patients use and understand the Open Payments database, and apply the information to their health care decision-making, could be valuable.

This study has several limitations. First, because of its observational design, this study’s findings may not necessarily indicate a cause-and-effect relationship. While difference-in-differences is a strong observational design, there may be unmeasured time-varying confounding that could explain our results, at least in part. Second, we used Medicare data and so our findings may not be generalizable outside of the Medicare population. Third, while we were able to link the year of prescriptions with the year of opioid-related payments, specific dates for each dispensed prescription were not available and therefore we could not determine if payments specifically preceded relevant prescriptions. Relatedly, data on opioid-related payments were not available for the first half of 2013 so it is possible that some physicians receiving payments that year were inappropriately included in the comparison group; however, this would bias our results toward the null. Fourth, a common identifier was not present for the Open Payments data and Medicare drug utilization data; while we used the best available matching techniques, we may have misclassified some physicians. However, a prior study using similar data found little difference in results using more and less stringent methods for matching [21]. Finally, we examined prescribing patterns at the physician level and had little clinical information about each physician’s patient panel. Determining appropriateness of opioid prescribing was not possible and changes in prescribing may represent a change in prescribing patterns among existing patients or reflect a change in the composition of a physician’s patient panel.

## Conclusion

Our analysis of a national cohort of physicians adds to the growing public policy concern that opioid drug manufacturers have worked to influence physician prescribing. Despite a low mean total dollar amount of opioid-related payments received, we found that prescribing significantly increased. Interventions are needed to either reduce such promotional activities or to mitigate their influence.

## Supporting information

S1 Table List of physician specialty categories for matching and analysis. (DOCX)

S2 Table (A) Distribution of Physicians by State Before and After Matching. (B) Distribution of Physicians by Specialty Before and After Matching. (C) Distribution of Physicians by Opioid Expenditures in 2013 Before and After Matching, for Cohorts of Physicians who Received Opioid-Related Payments in 2014 and 2015, but not in 2013. (D) Distribution of Physicians by Level of Opioid Expenditures Before and After Matching, for Cohorts of Physicians who Received Opioid-Related Payments in 2015, but not in 2013 and 2014.
(DOCX)

S3 Table Results of sensitivity analysis to test parallel trends assumption. (DOCX)

S4 Table Results of sensitivity analysis using expenditures for dispensed antibiotic drugs. (DOCX)
