# Supplementary material for: Payments from drug companies to physicians are associated with higher volume and more expensive opioid analgesic prescribing
Source: PLoS One. 2018 Dec 19;13(12):e0209383. doi: 10.1371/journal.pone.0209383 (PMC6300290; doi:10.1371/journal.pone.0209383)
Supplement: S1 Table — (DOCX) [file pone.0209383.s001.docx]

**S1 Table. List of Physician Specialty Categories for Matching and Analysis**

| **Addiction Medicine & Psychiatry** | **Non-Oncology Surgical Subspecialty** |
| --- | --- |
| Addiction Medicine | Cardiac Surgery |
| Neuropsychiatry | Colorectal Surgery (formerly proctology) |
| Psychiatry | General Surgery |
| **Anesthesiology and Pain Management** | Hand Surgery |
| Anesthesiology | Neurological Surgery |
| Interventional Pain Management | Neurosurgery |
| Pain Management | Obstetrics/Gynecology |
| **Dentist** | Ophthalmology |
| Dentist | Orthopaedic Surgery |
| Oral Surgery (dentists only) | Orthopedic Surgery |
| **Diagnostic Radiology and Interventional Radiology** | Otolaryngology |
| Diagnostic Radiology | Peripheral Vascular Disease |
| Interventional Radiology | Plastic Surgery |
| Nuclear Medicine | Plastic and Reconstructive Surgery |
| **Hospice and Palliative Care** | Thoracic Surgery |
| Hospice and Palliative Care | Urology |
| **Hospital-Based Non-Surgical** | Vascular Surgery |
| Critical Care (Intensivists) | **Oncology Medical Specialty** |
| Emergency Medicine | Hematology |
| Hospitalist | Hematology/Oncology |
| **Neurology** | Medical Oncology |
| Neurology | Radiation Oncology |
| Psychiatry & Neurology | Gynecological/Oncology |
| **Non-Oncology Medical Specialty** | Surgical Oncology |
| Allergy/Immunology | **Physical Medicine & Rehabilitation and Sports Medicine** |
| Cardiac Electrophysiology | Neuromusculoskeletal Medicine, Sports Medicine |
| Cardiology | Physical Medicine & Rehabilitation |
| Dermatology | Physical Medicine and Rehabilitation |
| Endocrinology | Sports Medicine |
| Gastroenterology | **Podiatry** |
| Infectious Disease | Podiatry |
| Interventional Cardiology | **Primary Care** |
| Nephrology | Family Medicine |
| Pathology | Family Practice |
| Pulmonary Disease | General Practice |
| Rheumatology | Geriatric Medicine |
| Sleep Medicine | Internal Medicine |
| Specialist | Osteopathic Manipulative Medicine |
|  | Pediatric Medicine |
|  | Preventive Medicine |
