# Supplementary material for: Payments from drug companies to physicians are associated with higher volume and more expensive opioid analgesic prescribing
Source: PLoS One. 2018 Dec 19;13(12):e0209383. doi: 10.1371/journal.pone.0209383 (PMC6300290; doi:10.1371/journal.pone.0209383)
Supplement: S2 Table — (A) Distribution of Physicians by State Before and After Matching. (B) Distribution of Physicians by Specialty Before and After Matching. (C) Distribution of Physicians by Opioid Expenditures in 2013 Before and After Matching, for Cohorts of Physicians who Received Opioid-Related Payments in 2014 and 2015, but not in 2013. (D) Distribution of Physicians by Level of Opioid Expenditures Before and After Matching, for Cohorts of Physicians who Received Opioid-Related Payments in 2015, but not in 2013 and 2014. (DOCX) [file pone.0209383.s002.docx]

**S2A Table. Distribution of Physicians by State Before and After Matching**

| **State** | **Distribution of Unmatched Physicians** | | |  | **Distribution of Matched Physicians for Cohort who Received Opioid-Related Payments in 2014 and 2015, but not in 2013** | |  | **Distribution of Matched Physicians for Cohort who Received Opioid-Related Payments in 2015, but not in 2013 and 2014** | |
| --- | --- | --- | --- | --- | --- | --- | --- | --- | --- |
|  | **Physicians who received Opioid-Related Payments in 2014 and 2015, but not in 2013** | **Physicians who received Opioid-Related Payments in 2015, but not in 2013 and 2014** | **Physicians that did not receive Any Opioid-Related Payment in Any Year** |  | **Physicians who Received Opioid-Related Payments** | **Physicians who did not Receive Any Opioid-Related Payment** |  | **Physicians who Received Opioid-Related Payments** | **Physicians who did not Receive Any Opioid-Related Payment** |
|  |  |  |  |  |  |  |  |  |  |
| Total | 6,432 | 9,503 | 271,473 |  | 6,322 | 191,478 |  | 8,669 | 153,723 |
| AK | 0.19% | 0.13% | 0.18% |  | 0.09% | 0.09% |  | 0.05% | 0.05% |
| AL | 3.09% | 2.53% | 1.64% |  | 3.08% | 3.08% |  | 2.34% | 2.34% |
| AR | 1.09% | 1.42% | 1.00% |  | 1.04% | 1.04% |  | 1.27% | 1.27% |
| AZ | 2.16% | 1.82% | 1.99% |  | 2.18% | 2.18% |  | 1.75% | 1.75% |
| CA | 8.30% | 8.54% | 10.91% |  | 8.43% | 8.43% |  | 9.03% | 9.03% |
| CO | 2.16% | 1.14% | 1.66% |  | 2.18% | 2.18% |  | 1.15% | 1.15% |
| CT | 1.62% | 1.24% | 1.03% |  | 1.63% | 1.63% |  | 1.17% | 1.17% |
| DC | 0.33% | 0.19% | 0.22% |  | 0.28% | 0.28% |  | 0.10% | 0.10% |
| DE | 0.34% | 0.31% | 0.27% |  | 0.30% | 0.30% |  | 0.20% | 0.20% |
| FL | 5.47% | 6.62% | 6.73% |  | 5.57% | 5.57% |  | 6.98% | 6.98% |
| GA | 2.39% | 3.67% | 3.03% |  | 2.34% | 2.34% |  | 3.81% | 3.81% |
| HI | 0.37% | 0.53% | 0.39% |  | 0.33% | 0.33% |  | 0.39% | 0.39% |
| IA | 0.92% | 0.75% | 0.93% |  | 0.85% | 0.85% |  | 0.70% | 0.70% |
| ID | 0.98% | 0.37% | 0.48% |  | 0.90% | 0.90% |  | 0.33% | 0.33% |
| IL | 3.11% | 3.18% | 3.76% |  | 3.16% | 3.16% |  | 3.31% | 3.31% |
| IN | 3.03% | 3.20% | 2.04% |  | 3.08% | 3.08% |  | 3.11% | 3.11% |
| KS | 1.49% | 1.47% | 0.87% |  | 1.47% | 1.47% |  | 1.45% | 1.45% |
| KY | 1.37% | 3.10% | 1.36% |  | 1.36% | 1.36% |  | 3.11% | 3.11% |
| LA | 1.41% | 2.52% | 1.65% |  | 1.41% | 1.41% |  | 2.43% | 2.43% |
| MA | 1.52% | 0.96% | 2.50% |  | 1.55% | 1.55% |  | 0.93% | 0.93% |
| MD | 2.08% | 1.60% | 1.61% |  | 2.06% | 2.06% |  | 1.49% | 1.49% |
| ME | 0.40% | 0.27% | 0.55% |  | 0.38% | 0.38% |  | 0.29% | 0.29% |
| MI | 4.48% | 4.15% | 3.86% |  | 4.54% | 4.54% |  | 4.22% | 4.22% |
| MN | 0.36% | 0.66% | 2.03% |  | 0.36% | 0.36% |  | 0.67% | 0.67% |
| MO | 3.30% | 3.21% | 2.08% |  | 3.27% | 3.27% |  | 3.25% | 3.25% |
| MS | 1.35% | 1.23% | 0.93% |  | 1.31% | 1.31% |  | 1.06% | 1.06% |
| MT | 0.44% | 0.24% | 0.34% |  | 0.41% | 0.41% |  | 0.18% | 0.18% |
| NC | 3.67% | 4.54% | 3.13% |  | 3.70% | 3.70% |  | 4.82% | 4.82% |
| ND | 0.12% | 0.20% | 0.24% |  | 0.11% | 0.11% |  | 0.16% | 0.16% |
| NE | 0.78% | 0.74% | 0.54% |  | 0.78% | 0.78% |  | 0.72% | 0.72% |
| NH | 0.37% | 0.33% | 0.47% |  | 0.35% | 0.35% |  | 0.28% | 0.28% |
| NJ | 3.37% | 3.33% | 2.61% |  | 3.39% | 3.39% |  | 3.09% | 3.09% |
| NM | 0.36% | 0.29% | 0.59% |  | 0.33% | 0.33% |  | 0.28% | 0.28% |
| NV | 0.84% | 0.98% | 0.72% |  | 0.84% | 0.84% |  | 0.85% | 0.85% |
| NY | 4.91% | 4.57% | 5.34% |  | 4.98% | 4.98% |  | 4.64% | 4.64% |
| OH | 4.73% | 3.39% | 3.94% |  | 4.79% | 4.79% |  | 3.46% | 3.46% |
| OK | 1.68% | 1.74% | 1.26% |  | 1.66% | 1.66% |  | 1.61% | 1.61% |
| OR | 1.00% | 0.78% | 1.57% |  | 0.98% | 0.98% |  | 0.76% | 0.76% |
| PA | 6.28% | 4.01% | 4.50% |  | 6.39% | 6.39% |  | 4.26% | 4.26% |
| PR | 0.11% | 0.44% | 1.33% |  | 0.09% | 0.09% |  | 0.40% | 0.40% |
| RI | 0.31% | 0.43% | 0.39% |  | 0.30% | 0.30% |  | 0.42% | 0.42% |
| SC | 1.99% | 3.39% | 1.58% |  | 1.95% | 1.95% |  | 3.45% | 3.45% |
| SD | 0.25% | 0.25% | 0.28% |  | 0.22% | 0.22% |  | 0.22% | 0.22% |
| TN | 2.50% | 3.74% | 2.23% |  | 2.52% | 2.52% |  | 3.82% | 3.82% |
| TX | 5.22% | 5.91% | 6.87% |  | 5.30% | 5.30% |  | 6.15% | 6.15% |
| UT | 1.07% | 0.62% | 0.72% |  | 1.03% | 1.03% |  | 0.63% | 0.63% |
| VA | 3.11% | 2.21% | 2.21% |  | 3.15% | 3.15% |  | 2.20% | 2.20% |
| VT | -- | 0.03% | 0.22% |  | -- | -- |  | 0.02% | 0.02% |
| WA | 1.41% | 1.09% | 2.35% |  | 1.39% | 1.39% |  | 1.15% | 1.15% |
| WI | 1.00% | 0.82% | 2.07% |  | 1.01% | 1.01% |  | 0.76% | 0.76% |
| WV | 1.03% | 1.09% | 0.63% |  | 1.01% | 1.01% |  | 1.00% | 1.00% |
| WY | 0.12% | 0.05% | 0.15% |  | 0.13% | 0.13% |  | 0.03% | 0.03% |

**S2B Table. Distribution of Physicians by Specialty Before and After Matching**

| **Specialty** | **Distribution of Unmatched Physicians** | | |  | **Distribution of Matched Physicians for Cohort who Received Opioid-Related Payments in 2014 and 2015, but not in 2013** | |  | **Distribution of Matched Physicians for Cohort who Received Opioid-Related Payments in 2015, but not in 2013 and 2014** | |
| --- | --- | --- | --- | --- | --- | --- | --- | --- | --- |
|  | **Physicians who received Opioid-Related Payments in 2014 and 2015, but not in 2013** | **Physicians who received Opioid-Related Payments in 2015, but not in 2013 and 2014** | **Physicians that did not receive Any Opioid-Related Payments in Any Year** |  | **Physicians who Received Opioid-Related Payments** | **Physicians who did not Receive Any Opioid-Related Payment** |  | **Physicians who Received Opioid-Related Payments** | **Physicians who did not Receive Any Opioid-Related Payment** |
|  |  |  |  |  |  |  |  |  |  |
| Total | 6,432 | 9,503 | 271,473 |  | 6,322 | 191,478 |  | 8,669 | 153,723 |
| Addiction Medicine & Psychiatry | 0.22% | 0.26% | 0.58% |  | 0.14% | 0.14% |  | 0.12% | 0.12% |
| Anesthesiology and Pain Management | 6.92% | 3.46% | 0.86% |  | 6.64% | 6.64% |  | 2.17% | 2.17% |
| Dentist | -- | -- | 9.44% |  | -- | -- |  | 0.08% | 0.08% |
| Diagnostic Radiology and Interventional Radiology | 0.05% | 0.06% | 0.10% |  | 0.02% | 0.02% |  | 0.01% | 0.01% |
| Hospice and Palliative Care | 0.02% | 0.02% | 0.06% |  | -- | -- |  | -- | -- |
| Hospital-Based Non-Surgical | 1.15% | 1.30% | 11.10% |  | 0.96% | 0.96% |  | 0.83% | 0.83% |
| Neurology | 4.09% | 6.60% | 1.40% |  | 3.73% | 3.73% |  | 5.27% | 5.27% |
| Non-Oncology Medical Specialty | 3.95% | 4.33% | 5.86% |  | 3.92% | 3.92% |  | 3.78% | 3.78% |
| Non-Oncology Surgical Subspecialty | 5.75% | 9.40% | 19.39% |  | 5.69% | 5.69% |  | 9.56% | 9.56% |
| Oncology Medical Specialty | 4.85% | 5.63% | 2.94% |  | 4.90% | 4.90% |  | 5.32% | 5.32% |
| Oncology Surgical Subspecialty | 0.16% | 0.22% | 0.28% |  | 0.14% | 0.14% |  | 0.16% | 0.16% |
| Physical Medicine & Rehabilitation and Sports Medicine | 4.24% | 3.24% | 1.22% |  | 4.07% | 4.07% |  | 2.20% | 2.20% |
| Podiatry | 0.31% | 1.05% | 2.16% |  | 0.30% | 0.30% |  | 0.97% | 0.97% |
| Primary Care | 68.30% | 64.34% | 44.61% |  | 69.47% | 69.47% |  | 69.52% | 69.52% |

**S2C Table. Distribution of Physicians by Opioid Expenditures in 2013 Before and After Matching, for Cohorts of Physicians who Received Opioid-Related Payments in 2014 and 2015, but not in 2013**

| **Level of Opioid Expenditures in 2013** | **Distribution of Unmatched Physicians** | |  | **Distribution of Matched Physicians for Cohort who Received Opioid-Related Payments in 2014 and 2015, but not in 2013** | |
| --- | --- | --- | --- | --- | --- |
|  | **Physicians who received Opioid-Related Payments in 2014 and 2015, but not in 2013** | **Physicians that did not receive Any Opioid-Related Payments in Any Year** |  | **Physicians who Received Opioid-Related Payments** | **Physicians who did not Receive Any Opioid-Related Payments** |
|  |  |  |  |  |  |
| Total | 6,432 | 271,473 |  | 6,322 | 191,478 |
| 62 - 1,484 | 10.00% | 59.30% |  | 10.14% | 10.14% |
| 1,484 - 3,454 | 10.00% | 13.01% |  | 10.16% | 10.16% |
| 3,454 - 6,132 | 10.00% | 8.11% |  | 10.14% | 10.14% |
| 6,132 - 9,463 | 10.00% | 5.61% |  | 10.11% | 10.11% |
| 9,463 - 13,746 | 10.01% | 4.24% |  | 9.90% | 9.90% |
| 13,746 - 19,432 | 10.00% | 3.21% |  | 10.08% | 10.08% |
| 19,432 - 27,927 | 10.00% | 2.53% |  | 9.92% | 9.92% |
| 27,927 - 40,949 | 10.00% | 1.77% |  | 9.87% | 9.87% |
| 40,949 - 67,790 | 10.00% | 1.27% |  | 9.81% | 9.81% |
| 67,790 - 1,096,354 | 10.01% | 0.96% |  | 9.89% | 9.89% |

**S2D Table. Distribution of Physicians by Level of Opioid Expenditures Before and After Matching, for Cohorts of Physicians who Received Opioid-Related Payments in 2015, but not in 2013 and 2014**

| **Level of Opioid Expenditures** | **Distribution of Unmatched Physicians** | |  | **Distribution of Matched Physicians for Cohort who Received Opioid-Related Payments in 2015, but not in 2013 and 2014** | |
| --- | --- | --- | --- | --- | --- |
|  | **Physicians who received Opioid-Related Payments in 2015, but not in 2013 and 2014** | **Physicians that did not receive Any Opioid-Related Payment in Any Year** |  | **Physicians who Received Opioid-Related Payments** | **Physicians who did not Receive Any Opioid-Related Payment** |
|  |  |  |  |  |  |
| Total | 9,503 | 271,473 |  | 8,669 | 153,723 |
| Distribution based on 2013 Opioid Expenditures | | | | | |
| 40 - 689 | 10.00% | 46.06% |  | 10.51% | 10.51% |
| 689 - 1,573 | 10.00% | 14.75% |  | 10.22% | 10.22% |
| 1,573 - 2,907 | 10.00% | 9.32% |  | 10.00% | 10.00% |
| 2,907 - 4,669 | 10.01% | 6.80% |  | 9.94% | 9.94% |
| 4,669 - 7,292 | 10.00% | 5.99% |  | 9.94% | 9.94% |
| 7,292 - 11,035 | 10.00% | 5.09% |  | 9.89% | 9.89% |
| 11,035 - 16,212 | 10.01% | 3.93% |  | 9.86% | 9.86% |
| 16,212 - 25,432 | 10.00% | 3.58% |  | 9.74% | 9.74% |
| 25,432 - 43,261 | 10.00% | 2.51% |  | 9.75% | 9.75% |
| 43,261 - 651,246 | 10.01% | 1.98% |  | 10.15% | 10.15% |
| Distribution based on 2014 Opioid Expenditures | | | | | |
| 53 - 441 | 5.00% | 31.18% |  | 5.32% | 5.32% |
| 441 - 858 | 5.00% | 15.56% |  | 5.26% | 5.26% |
| 858 - 1,343 | 5.00% | 8.52% |  | 5.17% | 5.17% |
| 1,343 - 1,976 | 5.00% | 6.56% |  | 5.19% | 5.19% |
| 1,976 - 2,740 | 5.00% | 5.05% |  | 5.10% | 5.10% |
| 2,740 - 3,588 | 5.00% | 4.11% |  | 5.05% | 5.05% |
| 3,588 - 4,520 | 5.01% | 3.37% |  | 4.96% | 4.96% |
| 4,520 - 5,763 | 5.00% | 3.44% |  | 5.02% | 5.02% |
| 5,763 - 7,134 | 5.00% | 2.97% |  | 4.95% | 4.95% |
| 7,134 - 8,879 | 5.00% | 2.92% |  | 4.90% | 4.90% |
| 8,879 - 10,904 | 5.00% | 2.54% |  | 4.99% | 4.99% |
| 10,904 - 13,066 | 5.00% | 2.13% |  | 4.99% | 4.99% |
| 13,066 - 15,836 | 5.00% | 2.07% |  | 4.82% | 4.82% |
| 15,836 - 19,440 | 5.01% | 1.98% |  | 4.84% | 4.84% |
| 19,440 - 23,964 | 5.00% | 1.73% |  | 4.88% | 4.88% |
| 23,964 - 30,666 | 5.00% | 1.71% |  | 4.91% | 4.91% |
| 30,666 - 39,524 | 5.00% | 1.32% |  | 4.83% | 4.83% |
| 39,524 - 52,629 | 5.00% | 1.02% |  | 4.83% | 4.83% |
| 52,629 - 78,578 | 5.00% | 0.88% |  | 4.91% | 4.91% |
| 78,578 - 877,375 | 5.01% | 0.93% |  | 5.05% | 5.05% |
