# Supplementary material for: Payments from drug companies to physicians are associated with higher volume and more expensive opioid analgesic prescribing
Source: PLoS One. 2018 Dec 19;13(12):e0209383. doi: 10.1371/journal.pone.0209383 (PMC6300290; doi:10.1371/journal.pone.0209383)
Supplement: S3 Table — (DOCX) [file pone.0209383.s003.docx]

**S3 Table. Results of Sensitivity Analysis to Test Parallel Trends Assumption**

|  | Mean Annual Expenditures for Dispensed Opioids Under Medicare Part D^*#^ | Mean Annual Daily Doses for Dispensed Opioids Under Medicare Part D^#^ | Mean Annual Expenditures per Daily Dose for Dispensed Opioids Under Medicare Part D^* #^ |
| --- | --- | --- | --- |
| **Payment-receiving physicians (n=8,669)** |  |  |  |
| 2013 | $21,930 | 10,167 | $2.19 |
| 2014 | $21,942 | 10,902 | $1.97 |
|  |  |  |  |
| **Comparison physicians (n=153,723)** |  |  |  |
| 2013 | $19,078 | 9,729 | $2.03 |
| 2014 | $19,227 | 10,399 | $1.84 |
|  |  |  |  |
| **Difference-in-differences estimate** | -$136 | 65 | -$0.03 |
| SE | *171* | *50* | *0.02* |
| 95% CI | *(-472 – 200)* | *(-33 - 163)* | *(-0.07 - 0.01)* |
| p-Value | *0.4271* | *0.1923* | *0.0924* |

Notes: * Adjusted for differences in prices over time ($ 2015). # Adjusted for average risk scores of beneficiaries treated by each provider.
