# Supplementary material for: Payments from drug companies to physicians are associated with higher volume and more expensive opioid analgesic prescribing
Source: PLoS One. 2018 Dec 19;13(12):e0209383. doi: 10.1371/journal.pone.0209383 (PMC6300290; doi:10.1371/journal.pone.0209383)
Supplement: S4 Table — (DOCX) [file pone.0209383.s004.docx]

**S4 Table. Results of Sensitivity Analysis using Expenditures for Dispensed Antibiotic Drugs**

|  | **Mean Annual Expenditures for Dispensed Antibiotics Under Medicare Part D^* #^** | |
| --- | --- | --- |
|  | **Physicians that Received Opioid-Related Payments in 2014 and 2015, but not in 2013** | **Analysis for Physicians that Received Opioid-Related Payments in 2015, but not in 2013 or 2014** |
| **Sample Sizes** |  |  |
| Payment-receiving physicians | 5,101 | 7,162 |
| Comparison physicians | 156,406 | 129,036 |
|  |  |  |
| **Payment-receiving physicians** |  |  |
| Pre-Intervention Period | $7,355 | 7,050 |
| Post Intervention Period | $6,917 | 6,506 |
|  |  |  |
| **Comparison physicians** |  |  |
| Pre-Intervention Period | $6,787 | 6,568 |
| Post Intervention Period | $7,868 | 5,952 |
|  |  |  |
| **Difference-in-differences estimate** | $229 | 72 |
| SE | *94* | *85* |
| 95% CI | *(45 - 414)* | *(-94 - 239)* |
| p-Value | *0.0150* | *0.3955* |

Notes: * Adjusted for differences in prices over time ($ 2015). # Adjusted for average risk scores of beneficiaries treated by each provider.
